# Preliminary Evaluation of a Personal Healthcare System Prototype for Cognitive eRehabilitation in a Living Assistance Domain

**DOI:** 10.3390/s140610213

**Published:** 2014-06-11

**Authors:** Matteo Pastorino, Alessio Fioravanti, Maria Teresa Arredondo, José M. Cogollor, Javier Rojo, Manuel Ferre, Marta Bienkiewicz, Joachim Hermsdörfer, Evangelia Fringi, Alan M. Wing

**Affiliations:** 1 Life Supporting Technologies, Universidad Politecnica de Madrid, Avenida Complutense 30, Madrid 28040, Spain; E-Mails: afioravanti@lst.tfo.upm.es (A.F.); mta@lst.tfo.upm.es (M.T.A.); 2 Centre for Automation and Robotics UPM-CSIC, Universidad Politecnica de Madrid, Calle de José Gutiérrez Abascal 2, Madrid 28006, Spain; E-Mails: jm.cogollor@upm.es (J.M.C.); javier.rojo.lacal@upm.es (J.R.); m.ferre@upm.es (M.F.); 3 Institute of Movement Science, Department of Sport and Health Science, Technische Universität München, Munich 80992, Germany; E-Mails: marta.bienkiewicz@tum.de (M.B.); joachim.hermsdoerfer@tum.de (J.H.); 4 School of Psychology, the University of Birmingham, Edgbaston, Birmingham B15 2TT, UK; E-Mails: EXF111@bham.ac.uk (E.F.); a.m.wing@bham.ac.uk (A.M.W.)

**Keywords:** cognitive rehabilitation, ambient assisted living, personal health system, eRehabilitation, remote rehabilitation

## Abstract

The integration of rehabilitation systems in an ambient assisted living environment can provide a powerful and versatile tool for long-term stroke rehabilitation goals. This paper introduces a novel concept of a personalized cognitive rehabilitation system in a naturalistic setting. The proposed platform was developed within the CogWatch project, with the intent of fostering independence in activities of daily living in patients with apraxia and action disorganization syndrome. Technical usability was evaluated in a series of pilot experiments, which illustrate how this approach may help to retrain patients in activities of daily living. The first system prototype has been tested with 36 participants divided into three groups, providing an exploratory evaluation of the usability of this solution and its acceptability. The technical solutions used within the CogWatch project are targeted to meet both the end users' needs from the interaction and usability point of views and the clinical requirements associated with the use of such systems. The challenges behind the development of ambient assisted living systems for cognitive rehabilitation are discussed.

## 1. Introduction

Stroke or cerebrovascular accident (CVA) is the second leading cause of death and disability in the population over 60 years old worldwide. Recently, it was estimated that around five million people each year suffer from a long-term physical and cognitive impairment due to stroke and require further rehabilitation to recover lost function [1]. Apraxic patients are susceptible to conceptual, spatial and temporal errors during activities of daily living (ADL) that can lead to potential health and safety issues (e.g., grasping a knife by the sharp edge) [2]. Difficulty with access to the relevant motor concepts is usually attributed to left brain lesions. Patients with right brain damage are often reported to suffer from action disorganization syndrome (ADS), which manifests in difficulty in performing ADL actions that involve many steps to achieve the goal (e.g., making a cup of tea or a sandwich). In apraxia and ADS, the inability to perform previously mastered activities in an independent way is a source of frustration for patients and puts significant burden on their carers [3].

### 1.1. Actual Rehabilitation Practice

The rehabilitation of apraxia and ADS remains a significant challenge for clinicians and therapists. There is still a scarcity of well-controlled research, and the results of available studies are not conclusive with respect to the effectiveness of the interventions proposed. One of the methods described by Goldenberg [4] consists of errorless training for apraxia patients with the assistance of the therapist, with each action step guided by a therapist and repeated. In this paradigm, the patient is prevented from committing errors in order to shape the correct behaviour and relearn motor concepts. This approach requires extensive training of each of the ADLs crucial for independence and needs to be tailored to other compromised abilities of the patient (for example, aphasia). The second approach that is also commonly practiced in the rehabilitation of stroke apraxia is explorative training, which allows the patient to explore freely the properties of the objects or tools. In this paradigm, patients are encouraged to infer the function of objects through the structure that will imply inference of the function of the objects through their structure. For rehabilitation of ADS, caused usually by right brain or frontal lobe damage, error minimizing strategies were proposed by Buxbaum *et al.* [5]. The first strategy is to remove all the objects that could potentially act as distractors in task performance [6]. The second strategy is based on verbal rehearsal techniques that help to create scripts for initiation and continuation of the task [7,8]. Many patients affected with apraxia also suffer from aphasia; therefore, some verbal-based techniques might be ineffective. However, even in those patients, some verbalization strategies, for example based on song recall, might be effective, as aphasia often spares singing. Bickerton *et al.* [9] presented a case study of an ADS patient who was taught a poem that prompted the individual steps in making a cup of tea. Results indicated that the sequencing of the actions improved whenever the patient used the song to guide his performance and correct errors. In another study [7], patients with apraxia were taught to verbalize their actions while performing ADLs. They found that verbalization led to improved ADL performance, compared to standard occupational therapy interventions. There is a consensus in the literature that multimodal cues and an individualized approach is necessary in the rehabilitation of apraxia and ADS. For in-depth reviews on the topic, please refer to Goldenberg [4] and Bienkiewicz *et al.* [10].

### 1.2. Hypothesis Definition

Goldenberg *et al.* [11] reported that consistent training improves specific task performance (food preparation), but cannot be generalized to other activities. Similarly, Donkervoort *et al.* [7] argued that intensive training usually brings a short-term benefit for most chronic patients who do not recover spontaneously. Current practice encompasses interventions that are based on retraining of ADLs using an errorless learning approach. Traditional approaches to cognitive rehabilitation for stroke survivors include the occupational therapist providing verbal or visual cues [12] while patients perform selected tasks. Training over a period of a few weeks post CVA provides the most promising outcome for future recovery. This type of service cannot be delivered, however, to all patients, due to the restricted funding for the post hospitalization phase in many countries. Moreover, this approach is labor intensive for therapy time, and there is limited opportunity for the patient to practice the skill. Therefore, technology-driven solutions are being developed to provide continuous ADL training and real-time support for the independence of stroke survivors. The use of information and communications technology (ICT) rehabilitation systems enables the training to be fully tailored to the needs of the patient in terms of the content of guidance (errorless training) and the modality of the information conveyed (auditory, visual, haptic outputs from the system). In addition, it allows shaping practice to the recovery rate in a similar way to the clinical setup with the occupational therapist. Within stroke therapy and the rehabilitation challenges, the EU funded project, CogWatch [13], proposes a personal healthcare system (PHS) to enhance the standard rehabilitation process of patients suffering from apraxia and action disorganization syndrome (AADS). It is characterized as being affordable, customizable and capable of delivering continuous cognitive rehabilitation at home [14]. The CogWatch system is being developed in relation to a set of scenarios involving ADL tasks. The system analyses the data coming from intelligent objects and gives multimodal feedback through speakers, vibro-tactile actuators and visual displays. This feedback guides the patient into correctly accomplishing the task [15]. This paper reports on an evaluation of the usability of the CogWatch system for cognitive eRehabilitation in a home environment and analyses the level of acceptance of a selected group of users.

## 2. Related Work

ICT has played an important role in rehabilitation during the last few years. A primary home-care debate is around whether sufficient qualified staff will be available if the ratio between the working age population and the elderly population changes [16]. ICT applied to healthcare systems has emerged as a suitable solution. In fact, many different technologies have been proven capable of coexisting with the medical environment [17]. An extensive review in cognitive rehabilitation confirms that there is no significant variation between using ICT solutions instead of traditional rehabilitation [18]. Furthermore, an updated version of this study highlights that in the particular case of AADS, strategy training is recommended, because it is more effective in improving ADL function than conventional occupational therapists (OT) [19]. ICT has not been only proven suitable for rehabilitation, but also useful for assessing impairments and diagnosing cognitive deficits. Based on speech recognition, an expert system could be useful for differentiating between dysarthria and apraxia of speech, which are complex disorders of speech that are produced by different neurological disturbances [20]. Any help to enhance the diagnosis of cognitive disorders involves the need for the proper rehabilitation of patients. Speech recognition has also been used to help in the diagnosis of different neural disorders, like autism [21]. According to Poizner *et al.*, recognition and signal processing of speech has the potential to support diagnosis, functional magnetic resonance imaging, trans-cranial stimulation, kinematic monitoring and modelling of neural motor disorders [22]. Nowadays, various commercial cognitive rehabilitation systems offer cognitive rehabilitation based on different solutions. Assistive robotics has evolved together with the emergence of different prototypes, but has mostly focused on motor skills recovery [23]. In addition, the use of cognitive robotics provided very useful solutions to therapies, such as the PARO (Paro Therapeutic Robot) [24] robot, which has been welcomed by psychologists for various therapies. With respect to day-to-day technology, mobile phones are considered suitable platforms thanks to their ubiquity and continuously improved accessibility [25–28]. A main issue related to these technologies is that elderly people are not always familiar with their use. Other attempts are based on immersive solutions that rely on virtual reality platforms and the use of rehabilitation games [29–31]. Even if such technologies involve high costs, the potential outcomes provide an optimistic prognosis for future developments in this direction [32]. The latest technologies create opportunities for human-computer interfaces that facilitate interaction with the patient through multiple feedback and monitoring devices, placed at patients' homes. Such solutions can be found in the COACH (Cognitive Orthosis for Assisting with Activities in the Home project) [33], based on hand washing task control, for patients having moderate-to-severe dementia. The major benefits of their approach include markerless motion tracking and the possibility to generalize to other ADL tasks [18]. A similar system, CoSy (Cognitive Systems for Cognitive Assistants) [34], prompts and messages to assist patients with the successful completion of tasks, directions, remembering schedules and other functions, providing feedback with a screen placed in a belt. Preliminary evidence shows that the system could help to contribute to successful rehabilitation, but it has not been assessed yet for a specific neural disorder [35]. Most common stroke rehabilitation systems, such as robotic arms and virtual environments, are focused on the physical rather than cognitive movement impairments of stroke patients. Among these are the MIMICS (Multimodal Immersive Motion rehabilitation with Interactive Cognitive Systems) [36] and ARMin (Arm Rehabilitation) [37] research projects, which assist the training of arm movements through, respectively, multimodal sensory feedbacks and cooperative control strategies.

Recent research solutions, such as REHAROB (Supporting Rehabilitation of Disabled Using Industrial Robots for Upper Limb Motion Therapy [38] and iPAM (Intelligent Pneumatic Arm Movement) [39] are designed to provide rehabilitation for people with hemiparesis. However, these solutions largely ignore the cognitive impairments associated with AADS and operate as station-platforms, which the patient has to access and to which the patient has to adapt. In contrast with these technological advancements, CogWatch intends to create an intelligent and complete assistance system, which will improve the activities of ADL in stroke survivors while living at home. A similar approach has been used in COACH [33] that aims to develop an intelligent system able to pervasively monitor older adults with Alzheimer's disease. However, the system has been designed for tracking basic human actions, hence being inadequate for more complex tasks, like ADL. Other research projects designed to assist patients with successful completion of ADL are ISAAC (Equipping People for Independence) [40] and GUIDE (Technology Indipendent Living) [41]. Both of the projects aim to deliver prompts to people with cognitive disabilities to help them complete specific tasks. A main drawback of such systems is the requirement of a constant user-machine interaction during the completion of a task. CogWatch minimizes the need for interaction between the user and the system as it monitors the progress of ADL tasks via sensorized objects.

The StrokeSleeve (Spatially Distributed Tactile Feedback for Stroke Rehabilitation) [42] research project, developed by the University of Pennsylvania (USA), aims at developing a low-cost sleeve that can measure the movement of the upper limb and assist the patient by providing tactile feedback at key locations. The feedback provided by the tactile system should guide the patient through a series of desired movements by allowing him or her to feel limb configuration errors at each instant in time.

Finally, DEM@CARE (Dementia Ambient Care) [43] and Contrast (Remote Control Cognitive Training) [44] aim at providing an adaptive human-computer interaction (HCI) that will improve impaired cognitive function through neurofeedback training.

Anyway, these solutions are not zero effort technologies (ZETs), since they require from the patient a period of training or do not provide automatic feedbacks (AF) that is delivered through a clinician during the visit.

A comparison of the various approaches herein collected is listed in Table 1. Common technical features include integrating the system in a home environment (HE), introducing ZETs, supporting cognitive rehabilitation (CR), executing complex tasks (CTs) and providing feedback based on automatic error recognition (AF). It shows the benefits and drawbacks of the current solutions.

The table underlines the need for a solution capable of offering a complete set of necessary features. Apart from StrokeSleeve, no other products or research projects present specific features for the cognitive rehabilitation of AADS users; for this reason, the CogWatch system has been designed to meet these requirements, indispensable for an adequate remote non-invasive rehabilitation. From the technical point of view, current projects tend to be underdeveloped in terms of usability, system maintenance and independent usage. For instance, with respect to the robotic-based solutions, therapists are required to spend a considerable amount of time programming the machines, monitoring the patients, analysing the data from the robot and assessing the progress of the patients. Such systems need to be programmed *ad hoc* for each individual rehabilitation case and cannot be executed remotely. With regard to the clinical validity of the previous projects, no positive effects have been clinically demonstrated in persons with AADS, and in some cases, no impact on performance on ADL has been observed after the rehabilitation period [45–47], since they are not tailored to the large variability of performance in AADS persons. In this regard, the CogWatch system has been designed for a wide range of AADS patients and, above all, to be configured and customized for a variety of rehabilitation settings.

## 3. Research Method

### 3.1. The CogWatch System Framework

**Figure 1.**
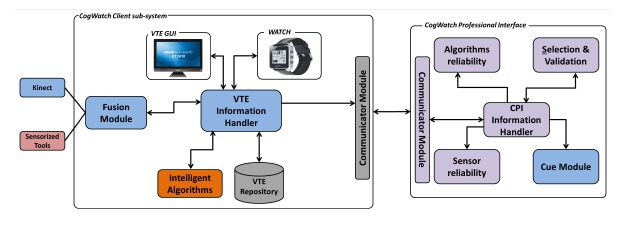
Architecture of the CogWatch first prototype.

Figure 1 shows the CogWatch design architecture. It consists of two main blocks: the CogWatch Client Sub-system (CCS) and the CogWatch Professional Interface (CPI).

The CCS, located at the patient's home, collects and analyses the data during the rehabilitation sessions. Within the CCS, two types of hardware devices have been considered, respectively for user monitoring and system feedbacks. The monitoring devices are designed to provide data from the patient's task execution and the corresponding movements and to enable the recognition of action errors. A Kinect™ from Microsoft and a set of sensorized objects were used for this purpose.

Kinect™ allows the system to track the user hand movements and behaviour when executing the task by providing the hands' position with a rate of 30 frames per second.

The device, equipped with two cameras and an infrared projector, can distinguish among different characteristics of the environment. The RGB cameras are used in order to get a color image for video recording of the workplace, while the infrared projector acquires depth information to deliver 3D positioning of both hands and the objects detected along the task execution.

The method of determining the 3D position for a given object or hand in the scene is determined through a triangulation process [48]. In order to ensure the correct implementation, it is necessary to calibrate the device, so that the compilation of the information results in being more accurate and reliable. The device is connected to the main system to process and store the coordinate data and video recordings.

Objects consisted of everyday tools, equipped with instrumented sensorized coasters. The instrumented coasters [49] are designed to provide data that can be used to recognize or analyse interactions with everyday objects, such as a mug, a jug or a kettle. A minimally invasive approach was employed to integrate sensors into the objects themselves, without interfering with their use or significantly modifying their appearance and without requiring any device to be fitted to the person using them. To achieve this goal, a wireless device that fits discreetly underneath the object was created. The sensors contained in the CogWatch Coaster include a 3-axis accelerometer and 3 force-sensitive resistors, together with a Bluetooth transmitter to send the data to the host computer.

The choice of small and low-cost sensors allows the sensorized objects to be inexpensive and easily used with other objects. Moreover, the sensors have low-power consumption and a rechargeable battery.

Apart from the monitoring, the feedback devices, composed of a desktop personal computer (PC) and a smart watch [50] interact with the patient and, when an error is detected, guide him through visual, auditory or vibro-tactile cues.

The PC monitor is equipped with a graphical user interface (GUI), named Virtual Task Execution (VTE), whose main objective is to provide the patient with the corresponding cues.

**Figure 2.**
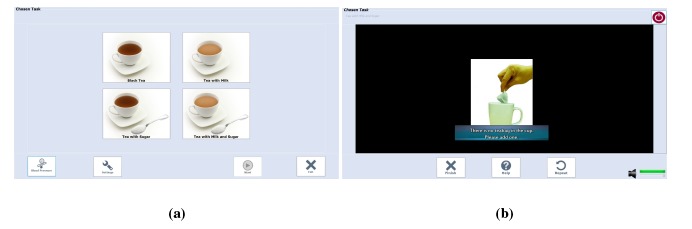
CogWatch selection and feedback screens. (**a**) CogWatch task selection screen; (**b**) CogWatch feedback screen.

The VTE GUI, shown in Figure 2a and Figure 2b, designed to interact directly with the users, was planned to be installed in both tablets and monitor screens, depending upon the users's preferences.

The watch, paired with the VTE, can be charged, programmed and debugged using a dedicated interface. The watch uses vibro-active feedback to warn the user about the forthcoming cue or action error.

The actions of the users are determined by an action recognition (AR) algorithm, able to identify human activities during their execution in a naturalistic scenario. After a pre-processing stage, the synchronized measurements from the sensors are transformed into a feature vector. The purpose of the AR is to transform the sequence of feature vectors, constructed from the outputs of the sensors, into the actions and sub-tasks performed by the patients.

CCS is comprised of software modules, focused on data handling (Virtual Task Execution (VTE) Information Handler), data synchronization (Fusion Module), data processing and error recognition (intelligent algorithms) and data storage (VTE Repository).

The CogWatch Professional Interface (CPI) represents the remote clinician side. It allows the professionals involved in the rehabilitation process to supervise the rehabilitation sessions with the real-time streaming video.

A communicator module is in charge of the CCS-CPI bi-directional communication.

**Figure 3.**
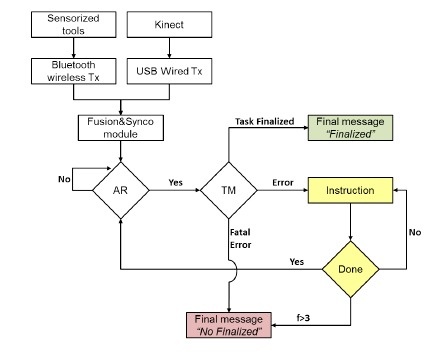
CogWatch workflow process.

Figure 3 shows the general workflow process of the CogWatch application. Signals are sent to the action recognition (AR) algorithm, after being pre-processed, synchronized and merged. Once an action has been identified by the AR, it is sent to a task model algorithm (TM). The purpose of the TM is to detect possible mistakes carried out by a subject and to estimate the next action to be performed in order to succeed with the action goal.

In this first evaluation phase, the AR algorithms are supervised by an expert to correct any possible discrepancy produced by the system.

In order to process time-varying sequences of data, a Markov decision process (MDP) has been adopted. Such algorithms are largely used in spoken language processing research as models of human interaction for dialogue processing [51]. The states of the MDP correspond to sequences of sub-goals that can be extended to a complete, successful instantiation of the tea making task. Associated with each state are a set of potential state transitions, corresponding to sub-goals, which are valid extensions of that state. When a sub-goal is output from the AR system, it is evaluated against these transitions. If the sub-goal corresponds to a valid extension of the current state, then the appropriate transition is taken, and the system moves into the corresponding new state. The data structure of the cue is a vector containing the ID of the cue that should be sent to the subject, its priority or its number and the next legal action predicted by the algorithm, if applicable.

Based on the MDP algorithm for decoding the sensors' continuous signals, the TM collates the sub-tasks identified by the AR and records the user's progress with respect to the task. The TM needs to be able to determine whether or not a particular set of sub-tasks is likely to result in the successful execution of the whole task. If the TM detects an error in the task execution, feedback is displayed to the user to guide the completion of the sub-task. If a non-recoverable error is detected, or the same error is detected more than 3 times, an error message suggests to the user to take a break and repeat the session later. Finally, when the TM detects that all sub-goals are accomplished, a successful completion message is shown. In Prototype 1.1, a tea preparation scenario has been selected as an example of a rehabilitation task. As shown in Table 2, different sub-actions are associated with the making of a specific type of tea.

### 3.2. Protocol Evaluation Methods

The main aim of CogWatch is to improve the quality of life of AADS people through personalized home rehabilitation. Due to the specific end user, the user friendliness (in clinical and usability aspects) has been a crucial aim of the user interface definition. Based on such requirements, specific goals were set:

- fit smoothly into the working or living routines of the users;
- user-friendly and easy to interact with.

Usability heuristics, guidelines and a standardized design methodology were employed to identify the usability issues in the prototype. User satisfaction was measured with questionnaires to identify potential areas of improvement of the system prototype. A table was used to simulate a typical kitchen table with the following utensils and implements placed on it:

- A mug for tea
- A tea spoon
- A sugar bowl
- A box containing tea bags
- A box to throw out the used tea bags
- A water jug
- A kettle, placed on a base kettle connected to the electricity supply
- A milk jug

Moreover, a touch screen PC was placed close to the participant in order to allow interaction with the system. Using these tools, the users were asked to prepare three types of tea, while one researcher was in charge of interacting with the users through the CPI interface. A second researcher collected all of the other information from the session and ensured the smooth recording of data from sensors. An *ad hoc* protocol was conceived of to test the developed system, following specified technical requirements, through user's interaction. The protocol was designed following the standard usability evaluation methods within generic system architectures. Nielsen (1993) states that such a test is “the most fundamental usability method and is in some way irreplaceable, since it provides direct information about how people use computers and what their exact problems are with the interface being tested” [52]. The chosen protocol is well suited to discover unexpected usability problems, which cannot be deduced from heuristics. This is especially important, as CogWatch is an innovative system, and hence, there are not any comparable systems from which to deduct additional knowledge. Finally, while requesting the repeated execution of the task was considered boring for the participants, this was necessary to get more information from a typical and usual task, in accordance with standard usability engineering [53,54].

Usability engineering is used to determine the user friendliness of a prototype. An important part of usability engineering is feedback from users, by working on typical tasks within the system. Following the same criteria, the aim was to determine how well the user interface supports the test participants. Users' behaviour was recorded, and their level of satisfaction was measured with questionnaires. Following these methods of analysis, it was possible to identify the final potential for the improvement of the software prototypes. The experiment has been based on two main phases: asking a user to interact with the CogWatch platform by executing a specified task and completion of the related usability questionnaire. The protocol consisted of the following steps:

(1) Prepare tea, by choosing among four types of tea (black tea, tea with milk, tea with sugar, tea with milk and sugar), the tools placed in the table set-up and the interactions with the VTE monitor.
(2) Repeat Step 1 twice, possibly by choosing a different tea with respect to the previous scenario.
(3) Usability questionnaire: participants are asked to fill in a questionnaire with a set of 22 statements.

The current version of the prototype was tested in three different institutions across Europe: Universidad Politecnica de Madrid (UPM), University of Birmingham (UoB) and Technical University of Munich (TUM). Since the CogWatch project is sensitive to ethical principles and data protection regulations at national, European and international level, participants were asked to formally agree on the recording, storage and processing of the information. The use of volunteer participants in the usability evaluation was approved by the ethical committee of the three institutions involved in the study (UPM, UoB and TUM).

Thirty-six users participated in the trials of the prototype. Participants were categorized into three main groups, according to their age and neurological condition. Twenty-seven of them were healthy (from UPM and TUM), while nine had AADS (from UoB and TUM). The mean age was 51.20 years. The gender and age distribution is shown in Table 3.

Even though the intervention group was not very copious and lower than the control one, it was sufficient to detect the majority of the usability issues. As defined by Nielsen [55], the best results in usability evaluation come from testing no more than five users and running as many small tests as possible. For this reason, the evaluation protocol of the CogWatch first prototype was based on multiple tests to improve the system usability and not only to document its weaknesses.

The tests conducted were focused on an extensive formative evaluation and on the summative analysis. The present usability study focused on the preliminary phase of design. Formative evaluation is focused on identifying usability problems before a product is completed. The summative evaluation describes the current usability of an interface in terms of task times, completion rates or satisfaction scores, assessing how usable an interface is [56]. Based on these concepts, the analysis of formative usability features was mainly acquired from healthy participants during the starting phase of the study. The aim was to verify whether the system had effectively fulfilled users' needs. The reason for using healthy participants for this phase was two-fold:

- Due to the severity of the condition of AADS patients, from an ethical point of view, the number of clinical participants involved was limited.
- In order to locate no obvious technical and design errors, it is necessary for the preliminary system to be tested on groups of diverse types of participants.

The feedback from patients was transformed into quantitative metrics with summative evaluations, in order to improve the design of the system and to refine the development specifications.

## 4. Results

**Figure 4.**
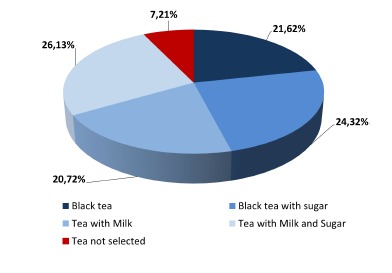
Tea task selection during the evaluation phase.

Three strands of evaluation activity have been coordinated across the pilot, involving stress tests of the software, system technical performance and system usability tests. The results of these evaluation activities have enabled the identification of a set of recommendations that will be used for the next version of the system. The first part of the evaluation was devoted to analysing the technical performance of the hardware and software of the system. A coherent and consistent approach to data collection was adopted across the stress tests and the system performance sessions in order to enable the integration and comparison of results across these two settings. During the evaluation phase, 111 sessions were recorded. The occurrences of tea selections are shown in Figure 4.

The audio cue message was selected nearly 80% of the time, while the text cue was selected only 20% of occasions. This result is linked to the users' limited attentional resources: participants preferred to focus on the task to be performed, rather than looking at the VTE monitor in case a cue appeared.

In 90.1% of the time, sessions were correctly recorded, even if some interaction errors were committed. More in detail, two types of errors were observed: the “finish” button was not pressed at the end of the session (NoFinish), and the system cue was not perceived by the participants during the session (NoCue). These issues did not affect the success of the rehabilitation sessions and were not equally distributed among them. The number and distribution of interaction errors among the groups is shown in Table 4. Sessions were not completed on 11 occasions. In particular, two sessions (1.8%) were aborted due to a failure of the Bluetooth connection that manages the communication between sensors and system; one session was not correctly finalized because the user (AADS) pressed the button “quit” instead of “finish” (Quit), at the end of the session. In 7.2% of the time, participants (three elderly, two young and three with AADS) started the session without selecting any tea type (NoTea).

High priority was given to the analysis of the behaviour of the external devices, consisting of Kinect™, the smart watch and the sensorized tools. The tests were based on observing the proper functioning of each device during the sessions. Kinect™ video camera recordings delivered robust data. Results show that 110 sessions were correctly stored in terms of video recording, tracking recording and streaming recording. Only one session was not correctly stored, because of a CPI-VTE communication error, however, not associated with the Kinect™. Regarding the smart watch, no particular issues were noticed on the vibration functioning during the sessions. In spite of this, the vibration intensity was considered too feeble, since not all of the patients were aware of it. Bluetooth connection robustness between VTE and the smart watch was also verified. Apart from a configuration problem observed on one session, results show the high stability of the system. Furthermore, the watch battery performance was tested, showing that, even if battery consumption was higher when the Bluetooth was turned on, no battery drain problems were observed. With respect to the sensorized tools (kettle base, kettle body, mug, milk jug), the sensor reliability, data storage and battery durability were reported for each user's session. The experiments indicate over 94% of the signals were correctly recorded during a test conducted with 36 participants.

In general, the CogWatch concept was widely accepted by the participants. Over 80% considered the system easy to use, and 85% found the overall look and feel of the GUI acceptable. Selected user comments are included below to indicate directions for further improvement.

### 4.1. Questionnaire Results (Participant Control)

After users completed the evaluation phase, a survey questionnaire was given to measure their satisfaction. The aim of the CogWatch Usability Test was to measure the usability of the prototype, in order to determine whether it was already acceptably high; and to identify potential improvements, in order to increase the usability of the CogWatch applications. The system usability test was based on the ISO 9241 standard that determines the quality of several usability aspects, such as:

- Effectiveness (can users successfully achieve their targets?);
- Efficiency (how much effort and resource are expended to achieve the proposed targets?);
- Satisfaction (was the experience satisfactory?).

The usability survey was based on the System Usability Scale (SUS), NASA Task Load Index (NASA-TLX) and AttrakDiff questionnaires [57–59], in accordance with the heuristic approaches described above. The surveys consisted of twenty-two statements, centered on evaluating the benefit of CogWatch and the appreciation of the participants by means of a 1–5 Likert scale. Thirty six users, from three different centers, participated in the survey. Among them, 27 users were healthy people, while nine were AADS patients. The analysis, resumed in Table 5, compares the results of the evaluation phase, in terms of mean (M) and standard deviation (SD). In order to evaluate possible statistical differences among groups, an independent, two-sample *t*-test has been applied, comparing the means of AADS/Elderlies samples (*p1*) and AADS/Youngs samples (*p2*). Through this test, it is possible to define if there is a correlation between the analysed samples (*p* > 0.05, not significant (ns)), or they are statistically different (*p* ≤ 0.05).

The first four statements were related to the usability analysis scale:
**Q1:**I think that I would like to use this system frequently.**Q2:**I thought the system was easy to use.**Q3:**I think that I would need the support of a technical person to be able to use this system.**Q4:**I think other people would find the system easy to learn.

**Figure 5.**
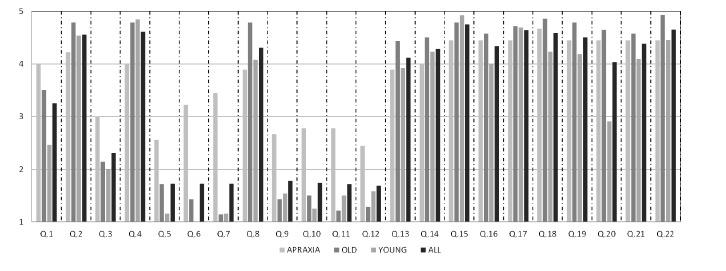
Questionnaire outcomes for the different study groups.

An analysis was conducted by calculating the weighted average for each statement's answer. The results of questionnaires for young, elderly and AADS patients are represented in Figure 5. The diagram shows, for each statement, Q, the average value according to five-level Likert scale items; where 1 represents the minimum perceived benefit and 5 the maximum perceived benefit. The outcomes of each user category are identified with a different colour.

On the whole, the analysis of the results reveals sizeable differences, in some case, among the groups of participants, especially between healthy people and patients. With respect to Statement Q1, a general level of satisfaction was perceived in the patients' willingness to use the system at home (M = 4, SD = 1.2). Results from Statement Q2 indicated that almost all of the participants consider the system easy to use (M = 4.5, SD = 0.7), although a considerable percentage of AADS patients solicited technical support on the use of the system at the beginning (M = 3.0, SD = 1.2) according to Q3. The answers from Statement Q4 denote that both elderly participants (M = 4.8, SD = 0.6) and AADS (M = 4.0, SD = 1.3) believed that other people would find the system easy to learn. In this case, the results revealed no significant differences between the groups (*p* > 0.05).

The second part of the survey referred to the system workload evaluation. The associated statements were:
**Q5:**The task was mentally demanding.**Q6:**The task was physically demanding.**Q7:**The pace of the task was hurried.**Q8:**I was successful in accomplishing what I was asked to do.**Q9:**I felt insecure during the test.**Q10:**I felt discouraged during the test.**Q11:**I felt stressed during the test.**Q12:**I felt annoyed during the test.

Mental workload, evaluated in Q5, was very low for the young (M = 1.2, SD = 0.4) and elderly (M = 1.7, SD = 1.4), while slightly arduous (M = 2.6, SD = 1.4) for AADS patients. Even so, its value can be considered acceptable. Physical workload was almost null for the young (M = 1.0, SD = 0) and for the elderly (M = 1.4, SD = 1.1). In contrast, patients reported a significant expenditure of physical effort (M =3.2, SD = 1.7) in performing the task, revealing statistical differences between the groups (*p* < 0.05).

With respect to the pace of the task performed, the analysis of Q7 showed that it was rushed for patients (M = 3.4, SD = 1.3), but not for other groups, with statistical differences between the samples (*p* < 0.05 in both cases).

Q8 revealed that almost all participants considered themselves successful in accomplishing what they were asked to do (M = 4.3, SD = 1.1). A comparison between the different groups revealed that there is no significant statistical differences in AADS and elderly samples (*p* > 0.05), in contrast with the AADS/young results (*p* < 0.05). A small degree of insecurity (M = 2.7, SD = 1.5), a lack of courage (M = 2.8, SD = 1.5), stress (M = 2.8, SD = 1.6) and annoyance (M = 2.4, SD = 1.4) were detected in AADS patients according to Statements Q9, Q10, Q11 and Q12. This might partly reflect the use of the system for the first time.

System attractiveness was evaluated through the following statements:
**Q13:**This system thinks the way I do.**Q14:**The communication between me and this system works very well.

Q15–Q22 were dedicated to evaluating the attractiveness of the task selection screen (Figure 2a) and the effectiveness of the feedback proposed (Figure 2b) when an error occurs. According to the answers on Statements Q13 and Q14, the participants' overall reaction was very satisfactory, with no statistical differences between the groups (*p* > 0.05 in both cases). Users considered the system well adapted to their needs (M = 4.1, SD = 0.9), and this was also true of communication (M = 4.3, SD = 1.0).

Statements Q15–Q22 were based on the reaction of the user looking at interface screen shots. Participants had to evaluate the screen between complicated/simple, ugly/attractive, dislike/like and confusing/clear. Results show that participants considered the system quite simple and pleasant to use (Q15–Q22 more than M = 4.0, 0.4 < SD < 1.1). Furthermore, many of them suggested the system should be more flexible to user conduct during the execution task. More than one fifth of the participants demanded an improvement in the look and feel of the graphic user interface.

According to the results, AADS patients considered themselves able to accomplish the task goal. They also agreed (Q20) that the system was easy and pleasant to use (M = 4.4, SD = 0.5) and, together with the older (M = 4.6, SD = 0.6, ns) ones, considered it more attractive than the younger (M = 2.9, SD = 0.8) ones.

Additional suggestions from the patients included adding a more descriptive figure of the selected tea, which would remain visible throughout the task. One of the participants suggested adding a confirmation sound when pressing a button, in order to receive a confirmation feedback from the system.

## 5. Conclusion

CogWatch represents a novel approach to the cognitive rehabilitation of people suffering from AADS, while staying in their own home environment, following the principles of commonly employed ambient assisted living paradigms. The aim of this preliminary study was to assess the usability of the system in terms of technical and usability outcomes. As the status of the system development is still in the prototype phase, the aim of the study was to provide directions for further improvement of the system, not validation of the system *per se*. Further investigation needs to focus on the correlation of clinical results with technical and usability results. In addition, we will in future compare the final prototype of this system with the state-of-the-art and provide the results of long-term usage. The experiments conducted with the first prototype of the CogWatch platform reveal that the system is adequately robust from a technical point of view. The usability evaluation provides evidence that the AADS patients can easily interact with the CogWatch system. Therefore, an extensive clinical validation phase will be conducted, with the objective of proving the rehabilitation benefits. The technical experiments on the hardware and software system's components indicate some deficiencies, primarily concerning the reliability of sensor communication. Such system vulnerabilities will be upgraded to improve system reliability in future versions. Further technical updates foresee the integration of a web portal interface for clinical user, allowing professionals to check the patients' rehabilitation session history remotely through a web interface. Moreover, professionals will be able to add and modify a patient data profile or schedule his/her rehabilitation sessions remotely. With respect to the assessment of usability, the methods used had a strong orientation towards not only end-user needs from the interaction point of view, but also the clinical requirements connected to the use of the system. According to patient feedback, the system helped avoid several errors in the tea making process, thanks to very helpful and clear video cues. Among them, 75% of them would favor using the system in their own home.

Such evaluation outcomes are valuable for the future of the CogWatch system. An important point is that end-users are neither designers nor technology experts, so that following their suggestions does not always lead to increased usability. However, such comments should be checked when developing the user interface. That is why these comments will be considered in further planning for the improvement of the CogWatch system.

## Figures and Tables

**Table 1.** Related works on stroke rehabilitation compared with the CogWatch.

**Project**	**HE**	**ZETs**	**CR**	**CT**	**AF**	**AADS**
**PARO**	YES	YES	NO	YES	NO	NO
**MIMICS**	NO	NO	YES	YES	NO	NO
**ARMin**	NO	NO	NO	YES	NO	NO
**REHAROB**	NO	NO	NO	YES	NO	NO
**COACH**	YES	YES	YES	NO	YES	NO
**ISAAC**	YES	YES	NO	NO	NO	NO
**GUIDE**	YES	NO	YES	YES	NO	NO
**StrokeSleeve**	NO	YES	YES	NO	YES	YES
**DEM@CARE**	YES	YES	YES	NO	NO	NO
**Contrast**	YES	NO	YES	NO	NO	NO

**CogWatch**	YES	YES	YES	YES	YES	YES

**Table 2.** Sub-actions for the tea making task.

**Sub-Action**	**Black Tea**	**Black Tea w/ Sugar**	**Tea w/ Milk**	**Tea w/ Milk and Sugar**
**Add water from jug to kettle**	YES	YES	NO	YES
**Boil water**	YES	YES	YES	YES
**Add teabag in cup**	YES	YES	YES	YES
**Add boiled water in cup**	YES	YES	YES	YES
**Add sugar into cup**	NO	YES	NO	YES
**Add milk**	NO	YES	YES	YES
**Stir**	YES	YES	YES	YES
**Remove tea bag**	YES	YES	YES	YES
**Toying with water jug**	YES	YES	YES	YES
**Toying with boiled water**	YES	YES	YES	YES
**Pressing finish button**	YES	YES	YES	YES

**Table 3.** Gender and age distribution during the trial.

**Group Type**	**Number of Users**	**Mean Age**	**Standard Deviation**	**Males**	**Females**
**> 60**	14	68.32	8.52	6	8
**< 60**	13	31.88	8.39	6	7
**AADS**	9	64.78	11.44	3	6

**TOTAL**	36	51.20	19.28	15	21

**Table 4.** Interaction errors distribution among groups.

**Group Type**	**Sessions**	**Interaction Errors**		**Fatal Interaction Errors**	

		**NoFinish**	**NoCue**	**Quit**	**NoTea**
**> 60**	42	9	6	–	3
**< 60**	39	3	1	–	2
**AADS**	30	16	9	1	3
**TOTAL**	111	28	16	1	8

**Table 5.** Statistical results of the different groups evaluated in the study.

	**AADS**		**Elderlies**			**Youngs**			**All**	
	**M**	**SD**	**M**	**SD**	***p1***	**M**	**SD**	***p2***	**M**	**SD**
Q1	4.0	1.2	3.5	1.7	ns	2.5	1.2	p ≤ 0.05	3.25	1.5
Q2	4.2	0.8	4.8	0.6	ns	4.5	0.8	ns	4.5	0.7
Q3	3.0	1.2	2.1	1.5	ns	2	1.4	ns	2.3	1.4
Q4	4.0	1.3	4.8	0.6	ns	4.8	0.4	p ≤ 0.05	4.6	0.8
Q5	2.6	1.4	1.7	1.4	ns	1.2	0.4	p ≤ 0.05	1.7	1.2
Q6	3.2	1.7	1.4	1.1	p ≤ 0.05	1	0	p ≤ 0.05	1.7	1.4
Q7	3.4	1.3	1.1	0.4	p ≤ 0.05	1.2	0.6	p 0.05	1.7	1.3
Q8	3.9	1.1	4.8	0.6	p ≤ 0.05	4.1	1.3	ns	4.3	1.1
Q9	2.7	1.5	1.4	1.1	p ≤ 0.05	1.5	0.8	p ≤ 0.05	1.7	1.2
Q10	2.8	1.5	1.5	1.2	p ≤ 0.05	1.2	0.6	p ≤ 0.05	1.7	1.2
Q11	2.8	1.6	1.2	0.4	p ≤ 0.05	1.5	1.2	p ≤ 0.05	1.7	1.3
Q12	2.4	1.4	1.3	0.6	p ≤ 0.05	1.5	0.9	p ≤ 0.05	1.7	1.1
Q13	3.9	0.9	4.4	0.8	ns	3.9	1.1	ns	4.1	0.9
Q14	4	1	4.5	0.7	ns	4.2	1.2	ns	4.3	1.0
Q15	4.4	0.5	4.8	0.4	ns	4.9	0.3	p ≤ 0.05	4.75	0.4
Q16	4.4	0.7	4.6	0.6	ns	4	0.8	ns	4.3	0.8
Q17	4.4	0.5	4.7	0.6	ns	4.7	0.5	ns	4.6	0.5
Q18	4.7	0.5	4.9	0.4	ns	4.2	0.9	ns	4.6	0.7
Q19	4.4	0.5	4.8	0.6	ns	4.1	1.1	ns	4.5	0.9
Q20	4.4	0.5	4.6	0.6	ns	2.9	0.8	p ≤ 0.05	4.3	1.0
Q21	4.4	0.5	4.6	0.8	ns	4.1	0.8	ns	4.4	0.7
Q22	4.4	0.5	4.9	0.3	p ≤ 0.05	4.4	0.8	ns	4.7	0.6
